# Opportunities and challenges for verbal autopsy in the national Death Registration System in Sri Lanka: past and future

**DOI:** 10.1186/1478-7954-9-21

**Published:** 2011-08-01

**Authors:** Samath D Dharmaratne, Rajitha L Jayasuriya, Buddhipani Y Perera, EM Gunesekera, A Sathasivayyar

**Affiliations:** 1 Department of Community Medicine, Faculty of Medicine, University of Peradeniya, Sri Lanka; 2 Registrar General's Department, Colombo, Sri Lanka

## Commentary

Analyses of cause of death (COD) statistics are fundamental for monitoring the health situation of populations and for planning suitable interventions. The accuracy of national COD statistics is important for reliably prioritizing health problems in order to decide upon interventions and for resource allocation. The completeness of registration in the Death Registration System (DRS), which is a major component of the Civil Registration System (CRS) of Sri Lanka, is above 90% [1]. However, the quality of COD statistics are deficient, with 30% of deaths categorized as being due to "signs, symptoms, and ill-defined causes" [2]. Deaths coded to these categories are of little use for decision-making. There is considerable potential for verbal autopsy to complement the DRS to improve the quality of COD statistics in Sri Lanka [3].

In Sri Lanka, once a death occurs, it has to be registered before the deceased can be cremated or buried. For deaths that occur outside hospitals, the relatives of the deceased notify the Death Registrar (DR). The majority of these notifications will not have a medically-determined COD, and the Death Registrar determines this by interviewing the relatives regarding events preceding the death.

For deaths that occur in hospitals, a COD is declared by the medical officer who attended the deceased by filing a Death Declaration Form (DDF). Except for "sudden deaths," the COD for three out of four deaths that occur outside a hospital is given by the Death Registrar. Sudden deaths (which are a small proportion of total deaths) that occur outside a hospital are attended by an Inquirer into sudden death or by a court of law. The majority of the Death Registrars are lay people with minimal or no training in how to decide on the probable COD. Deaths that occur outside a hospital, in the majority of instances, do not have a death declaration made by a medical officer (A.Sathasivayyar. Assistant Registrar General of Sri Lanka. 3-11-2010 - personal communication).

Several studies conducted in Sri Lanka [1,4], have highlighted the biases that are present in the DRS. They point out that only 30% to 40% of the registered deaths occur in a government hospital and that 80% of the registration and certification of deaths is done by nonmedical registrars. A study carried out in 1996 to assess the quality and coverage of death certification found that 15.5% of the medical officers misclassified the underlying cause of death. The study also found that the use of ill-defined terms (e.g., cardiovascular arrest) was frequent (76.4%), as was the use of abbreviations leading to misclassification (26.4%) [1].

The Verbal Autopsy Questionnaire (VAQ), introduced into Sri Lanka in 2006, has several important limitations: only a limited number of diseases are included that encompass very broad categories, such as high blood pressure, heart disease, diabetes, kidney disease, paralysis, wheeze, any fever and cancer; symptoms asked about are limited; and the ability of the Death Registrars to identify the correct disease using this VAQ is also limited.

Nonetheless, the introduction of a VAQ with the support of policymakers is an important step towards improving the quality of cause of death data in Sri Lanka. This not only has sensitized the government to the technique of verbal autopsy but users (i.e., Death Registrars) now accept it as an integral part of their function in certifying deaths and as an important step towards improving data quality. The difficult part of the policy change, establishing the system and ensuring that the Death Registrars accept it, has already been achieved. What is now needed is to improve it: to restructure and expand the VAQ to include additional items to help the Death Registrars to arrive at a probable COD using the VAQ.

The Standard VAQ developed by the World Health Organization (WHO), which is also being used in a number of other countries for improving COD statistics, is being translated and validated for routine use in Sri Lanka. This innovation, particularly if combined with automated methods for diagnosing cause of death, has the potential to substantially improve the quality and timeliness of critical cause of death information for policy and planning in Sri Lanka.

## Competing interests

The authors declare that they have no competing interests.

## Authors' contributions

SDD planned the study and data collection, analyzed and interpreted the data, and prepared the manuscript. RLJ helped in the data acquisition and helped in the preparation of the manuscript. The other authors were involved in acquisition of data and manuscript preparation.
